# Supplementary material for: N, S Co-Doped Carbons Derived from Enteromorpha prolifera by a Molten Salt Approach: Antibiotics Removal Performance and Techno-Economic Analysis
Source: Nanomaterials (Basel). 2022 Dec 2;12(23):4289. doi: 10.3390/nano12234289 (PMC9737878; doi:10.3390/nano12234289)
Supplement: Supplementary file 1 [file nanomaterials-12-04289-s001.zip › nanomaterials-2050507-supplementary.pdf]

Supporting information

N, S Co-Doped Carbons derived from *Enteromorpha prolifera* by a Molten Salt Approach: Antibiotics Removal Performance and Techno-Economic Analysis

Mengmeng Zhang [1], Kexin Huang [2], Yi Ding [1], Xinyu Wang [2], Yingli Gao [1], Pengfei Li [1,*], Yi Zhou [2], Zheng Guo [3], Yi Zhang [3] and Dapeng Wu [2,*]

[1] School of Business, Henan Normal University, Xinxiang 453007, China; zhangmengmeng@htu.edu.cn (M.Z.); dingyihnu@163.com (Y.D.); gaoyinglihtu@163.com (Y.G.)
[2] Key Laboratory of Green Chemistry Medias and Reactions, Ministry of Education, School of Environment, Henan Normal University, Xinxiang 453007, China; ke1919124086@163.com (K.H.); wangxinyuhtu@163.com (X.W.); zhouyihtu@163.com (Yi Zhou.)
[3] College of Textiles, Zhongyuan University of Technology, Zhengzhou 451191, China; huiyi1981@163.com (Z.G.); zhangyihtu@163.com (Yi Zhang)
* Correspondence: lipengfei@htu.edu.cn (P.L.); dapengwu@htu.edu.cn (D.W.)

1.. . Experimental details

1.1. Preparation of stock solution

Sulfamethoxazole solution (SMX): 0.2 g of sulfamethoxazole was dissolved in 2000 mL distilled water with ultrasound for 24 hours (2000 mL volumetric flask). Potassium persulfate solution (PDS): 6.75g potassium persulfate was dissolved in 250 mL distilled water, to prepare 50 mmol L^{-1} stock solution.

1.2. Effects of each component in the AOPs

Firstly, three 250 mL conical flasks, numbered 1, 2 and 3, were respectively filled with 50 mL SMX solution, then added with 50 mL deionized water. For flask 1, 0.05g bio-carbon was added without adding PDS solution. For flask 2, 0.05g bio-carbon was added and then added with 10mL 50mmol L^{-1} PDS solution. For flask 3, 10 mL 50mmol L^{-1} PDS solution was added without bio-carbon. The flasks were placed on a shaking table with a rotation speed of 180 r min^{-1}. The sampling time was set as 5, 10, 30, 60, 65, 70, 90, 120 and 180min. Before filtered with 0.22 μm filter in each sampling, 100 μL methanol was added to quench the reaction. After diluted three times, the samples were tested by UV spectrophotometer at 260 nm. The measured absorbance was fitted using the standard curve (y=0.508x+0.0346) to calculate the corresponding concentration and removal rate. All the testing results are averaged by three times.

1.3. Effect of different reaction conditions on the AOPs

The pH of the SMX solution was adjusted to 2, 4, 6 and 9 with diluted HCl or NaOH. The absorbance is obtained according to the above testing and sampling methods, and the removal rate after 120 min is calculated based on the standard curve to study the effect of different pH on the AOPs.

0.025 g, 0.05 g, 0.075 g and 0.1 g EBC-K were respectively into the SMX solution to conduct the above advanced oxidation experiments to study the effect of EBC-K dosages. in order to study the degradation rate of EBC-K in SMX solutions with different concentrations, the SMX solution was also diluted to 20 mg L^{-1}, 30 mg L^{-1}, 40 mg L^{-1}, 50 mg L^{-1} and 60 mg L^{-1}, 0.05 g of EBC-K was added respectively to start the AOPs process according to the above steps.

1.4. Actual water treatment

In order to investigate the performance of EBC-K in real water treatment, running water fetched from the local water supply company (Xinxiang City, Henan Province of

China) was used as the background to prepare the 50 mg L^{-1} SMX solution. 0.05 g EBC-K was added into the SMX involved running water, and the AOPs were carried out in a similar methods.

2. Details for the calculation of the COM

The cost of manufacture (COM) is composed of five main parts: the Fixed capital investment (FCI), costs of labor (C_{OL}), raw materials (C_{RM}), waste treatment (C_{WT}) and utilities (C_{UT}). According to Nontipa Supanchaiyamat's work, the COM of the bio bio-carbon can be calculated with the following equation:

$$COM = 0.230FCI + 2.73C_{OL} + 1.23(C_{RM} + C_{WT} + C_{UT})$$

2.1. FCI

The price of the industrial furnace is $ 3,500 and the mixing machine is $ 3,000. Four groups of furnaces and mixing machines are installed in the factory, so the total FCI is $26,000.

2.2. C_{OL}

The local average wage in Shandong Province is $ 3.57 h^{-1} (National Bureau of Statistics of China, 2021)[2]. The factory works 330 days a year, carries out 24-hour shift and requires four employees per shift. Based on this, the furnaces and mixing machines will operate for 7,920 h per year and the labour cost is $ 28.56 per hour. Therefore, the total annual C_{OL} is $ 226,195.2.

2.3. C_{RM}

The raw material costs include:

(i) EP

The price of EP is $ 9.17 $tonne^{-1}$ [3]. The capacity of the furnace is ~3,000 L, the density of the EP is 0.35 g cm^{-3}. Therefore, the furnace will process ~1000 kg biomass each time. Also, the mixing mashing is also adapted to the furnace. Based on this calculation, the facility can process about 5,280 batches annually. The total cost of the biomass is $ 48,417.6 per year.

(ii) Salt

The price of NaCl and KCl is $ 80 and $ 260 per tonne respectively [4,5]. As the weight ratio of EP biomass and salt used in the molten salt process is 1:1, 5,280 tonnes of the biomass are used every year. Plus,1/2 of biomass can be recycled. Based on this,2,640 tonnes of salt will be needed and the total cost of KCl and NaCl are $ 686,400 and $ 211200 per year respectively.

In summary, the total C_{RM} adds up to $ 734,817.6 and $ 259,617.6 per year respectively.

2.4. C_{WT}

Water and salt (about 1/2 of the total salt) can be recycled, which makes the C_{WT} can be ignored.

2.5. C_{UT}

The furnaces are operated at the maximum power of 150 kw and the mixing machines at the maximum power of 30 kw. The cost of industrial and commercial electricity price in Shandong Province is $0.099 h^{-1} [6], so all machines will consume $ 17.82 h^{-1}. Since four groups of furnaces and mixing machines will be used, the cost will be $ 71.28 h^{-1}. Therefore, the total annual cost is $ 564,357.6.

The residual heat from the furnace will be recycled to dry the biomass, which leads to the costs of drying process is negligible.

Therefore, the total COM could be calculated as following (the bio bio-carbon yield is assumed as 18%):

$$\mathrm{COM(KCL)} = 0.230(26{,}000) + 2.73(226{,}195.2) + 1.23(734{,}817.6 + 0 + 564{,}357.6)$$

$$\mathrm{COM(NACL)} = 0.230(26{,}000) + 2.73(226{,}195.2) + 1.23(259{,}617.6 + 0 + 564{,}357.6)$$

$$\mathrm{COM(KCL)} = \frac{0.230(26{,}000)+2{,}73(226{,}195.2)+1.23(734{,}817.6+0+564{,}357.6)}{5{,}280(18\%)} = 2.43\ \ (\$)$$

$$\mathrm{COM(NACL)} = \frac{0.230(26{,}000)+2{,}73(226{,}195.2)+1.23(259{,}617.6+0+564{,}357.6)}{5{,}280(18\%)} = 1.72\ \ (\$)$$

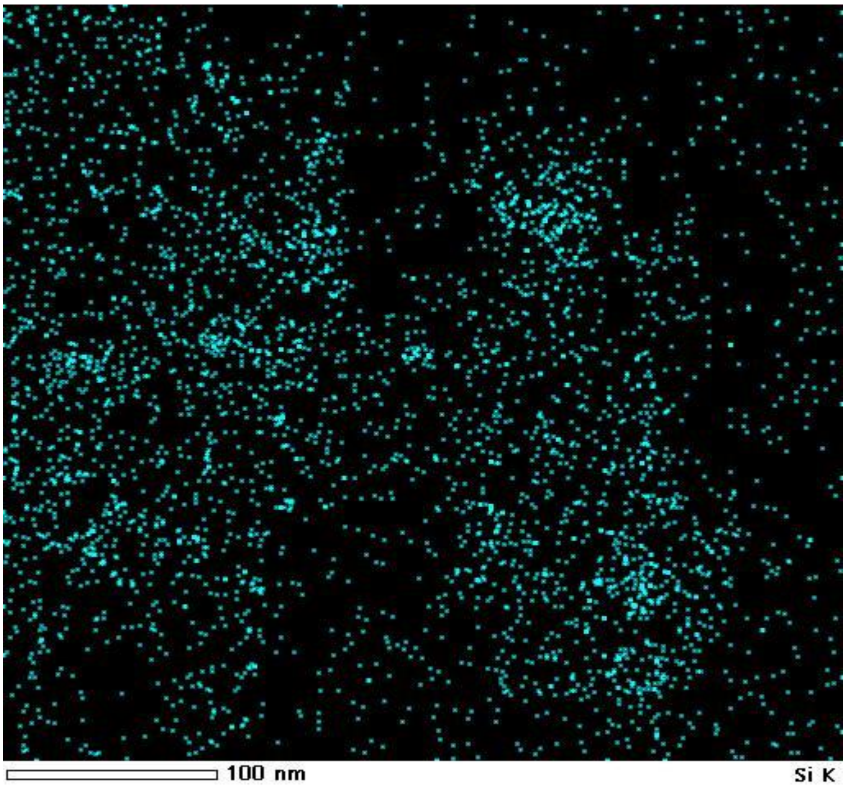

Figure S1. The Si distribution of EBC-K by HAADF-STEM and EDS mapping.

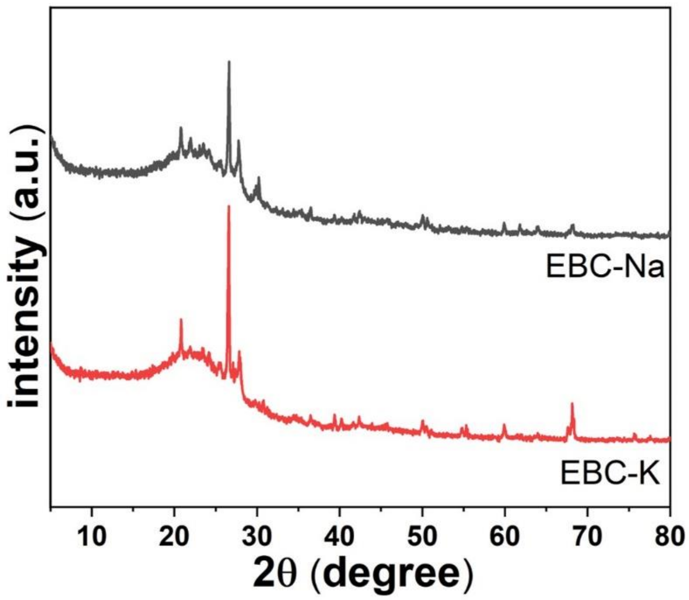

Figure S2. XRD curves of the EBC-Na and EBC-K.

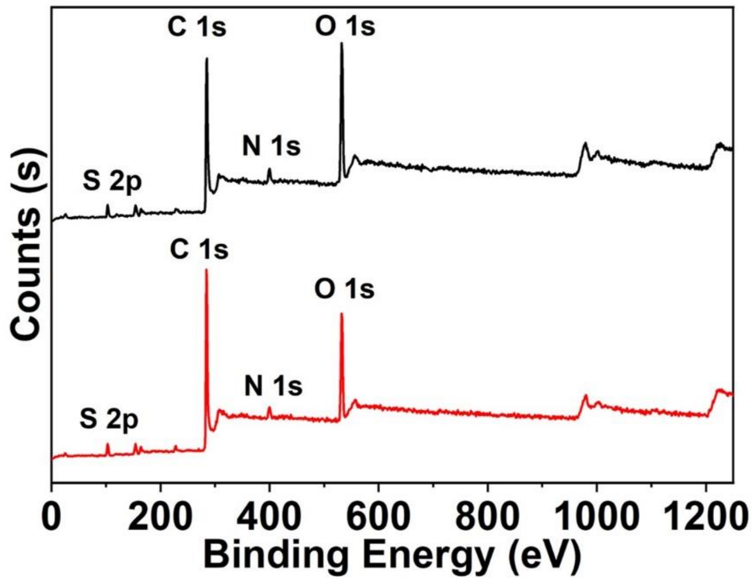

Figure S3. The XPS survey curves of EBC-K (black) and EBC-Na (red).

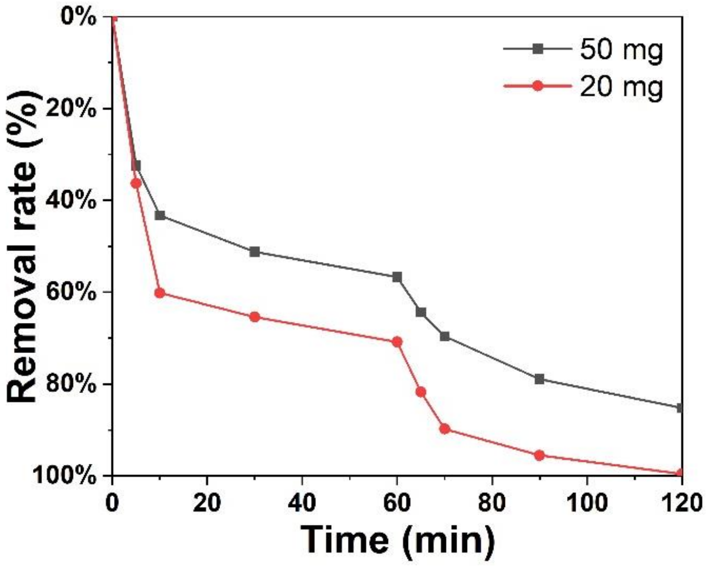

Figure S4. SMX degradation in pure water and practical water treatment (local running water as background).

Table S1. the XPS results on the distribution of the C, N, O and O elements.

**Sample**	**Element content (atm %)**			
	**C**	**N**	**O**	**S**
EBC-K	68.73	4.85	24.07	2.35
EBC-Na	74.18	4.77	19.09	1.96

Supporting references

1. Sangon, S.; Hunt, A.J.; Attard, T.M.; Mengchang, P.; Ngernyen, Y.; Supanchaiyamat, N. Valorisation of waste rice straw for the production of highly effective carbon based adsorbents for dyes removal. *J. Clean. Prod.* **2018**, *172*, 1128–1139. https://doi.org/10.1016/jjclepro.2017.10.210.
2. National Bureau of Statistics of China. 2021. Available online: http://www.stats.gov.cn/tjsj/ndsj/2021/indexch.htm (accessed on 15 October 2022).
3. Lalamove. Available online: https://m.huolala.cn (accessed on 1 November 2022).
4. Zouping Changshan Town Zefeng Fertilizer Factory. 2020. Available online: https://www.alibaba.com/product-detail/food-grade-99-99-purity-potassium_60225947884.html?spm=a2700.galleryofferlist.normal_offer.d_title.f9be2c9fkqJoD6 (accessed on 1 February 2020).
5. Shouguang Hengyi Chemical Technology Co., Ltd. 2020. Available online: https://www.alibaba.com/product-detail/Sodium-Chloride-NaCL-99-3-Industrial_60571951782.html?spm=a2700.galleryofferlist.normal_offer.d_title.48187c45LVfvGC (accessed on 1 February 2020).
6. State Grid Shandong Electric Power Company. 2022. Available online: http://www.sd.sgcc.com.cn/html/main/col2752/2022-10/09/20221009083047270522735_1.html (accessed on 15 October 2022).
